# Different types of tumor vessels in breast cancer: morphology and clinical value

**DOI:** 10.1186/s40064-015-1293-z

**Published:** 2015-09-17

**Authors:** Marina A. Senchukova, Natalia V. Nikitenko, Olesia N. Tomchuk, Nikon V. Zaitsev, Alexander A. Stadnikov

**Affiliations:** Department of Oncology, Orenburg State Medical University, Orenburg, Russia; City Oncology Clinic, No 2, Krasnodar Region, Russia; Department of Histology, Cytology and Embryology, Orenburg State Medical University, Orenburg, Russia; Orenburg Regional Clinical Oncology Center, Orenburg, Russia

**Keywords:** Breast cancer, Angiogenesis, Tumor vessels

## Abstract

Angiogenesis is a key factor of tumor progression. Considering, that the tumor vessels are heterogeneous and differ in morphology and clinical significance, the purpose of this research was to study of the morphological features of tumor vessels and their relationship with the clinical characteristics and morphological features of breast cancer (BC). In this pilot study the tumor samples received from 59 patients with T1–T2 stages of ductal invasive carcinomas were included. The sections were stained with hematoxylin and eosin and immunohistochemically using antibodies to CD34. The morphological features and the number of different types of tumor vessels were assessed microscopically and were compared with grade, lymph node metastasis, hormone receptors, HER2/neu status and with the presence of tumor emboli in vessels (lymphovascular invasion). We identified the following types of tumor vessels in BC: the normal microvessels, the dilated capillaries of peritumoral stroma, the atypical dilated capillaries and the “cavitary” structures (CS) type-1 and type-2 relating to the “cavitary” type of angiogenesis that was described by us earlier. The number of dilated capillaries correlated with CS type-1 (p = 0.003), CS type-2 (p = 0.002), atypical dilated capillaries (p = 0.0008) and with lymphovascular invasion (p = 0.005); the presence of atypical dilated capillaries—with CS type-1 (p < 0.00001), CS type-2 (p = 0.00004), lymphovascular invasion (p = 0.0002) and with the tumor grade (p = 0.003); the number of CS type-1—with estrogen receptor (p = 0.002) and progesterone receptor (p = 0.002) status and with lymphovascular invasion (p = 0.004); the presence of CS type-2—with positive Her2/new status (p = 0.0002) and lymphovascular invasion (p = 0.01). The density of normal microvessels was not associated with other types of tumor vessels and with clinical characteristics of BC. These data indicate that varied types of tumor vessels are associated with different morphological characteristics of BC, such as hormone receptors and HER2/neu status, lymphovascular invasion. We believe that the atypical dilated capillaries are related to the “cavitary” type of angiogenesis. The strong correlations of lymphovascular invasion with CS type-1 and atypical dilated capillaries testify that the “cavitary” type of angiogenesis may play a significant role in the formation of tumor emboli in the vessels.

## Background

Breast cancer (BC) is the leading cause of cancer deaths among women. The prognosis of BC treatment results is a serious problem since a variety of clinical, morphological, molecular-biological and molecular-genetic factors influences the behavior of tumor cells and their response to the treatment. These factors include patient age, histological type and grade, tumor size, lymph node status, estrogen receptor (ER) and progesterone receptor (PR) status, and human epidermal growth factor receptor 2 (HER-2) status (Fitzgibbons et al. 2000; Kalaja 2007; Lester et al. 2009). Although adjuvant treatment is being individualized according to these factors, not all patients show the same response to the treatment. In this respect the knowledge of the prognostic factors is important for predicting recurrence and metastasis and for choosing target therapies.

One of the most significant factors of tumor progression is angiogenesis (Folkman 1998). However, its role in the progression of BC has been studied insufficiently and the received data are contradictory (Uzzan et al. 2004). For example, several studies have indicated that a high microvessel density (MVD) in tumor is associated with more advanced stages and poor prognosis of BC (Popiela et al. 2008; Fernández-Guinea et al. 2013; Li et al. 2013). At the same time, the other authors have found no association of MVD with clinical characteristics and long-term results of BC treatment (Fridman et al. 2000; Gasparini 2001; Mohammed et al. 2013). Even more, the individual researchers have shown that some blood vessels presented within the tumor microenvironment can be associated with a favorable prognosis (Martinet et al. 2013). We believe that the noted contradictions may be due to the fact that in most researches the study of the angiogenesis in BC has been carried out without the considerations of the tumor vessels features. This approach is not quite justified since it is known that the blood vessels in tumors are heterogeneous and differ both in origin and morphology as well as in clinical significance (Fukumura et al. 2010; Birau et al. 2012; Nagy and Dvorak 2012; Martinet et al. 2013).

We have previously described a new way of angiogenesis, named by us the “cavitary” type of angiogenesis, and have showed its clinical significance on the example of gastric cancer (GC). This type of angiogenesis consists in the formation of “cavitary” structures (CS) in the tumor stroma and adjacent gastric mucosa, being then lined by the endothelium and merged into the blood vessels of the organ (Senchukova and Kiselevsky 2014). The obtained data have allowed us to propose the existence of two main mechanisms of CS formation. The first one is associated with the abruption of layers of epithelial cells from their underlying foundation and their desquamation into the lumen of the “obliterated” tumor glands, the second—with the formation of CS directly in the tumor stroma or adjacent tissue. In our study the presence of multiple CS type-1 was associated with the T3-4 and N2 stages as well as with the decrease of long-term results of GC treatment. The presence of CS type-2 was only associated with the diffuse type of GC.

Considering the difference of the contribution of various types of vessels in the tumor progression, the purpose of this research was to study the morphological features of tumor vessels and their relationship with the clinical and morphological features and with the tumor receptor status of BC.

## Methods

### Patients

Fifty nine patients with invasive ductal BC who had undergone surgery between May 2011 and April 2013 at the Orenburg Regional Clinical Oncology Center, were included in this pilot study. The study was performed in accordance with the Helsinki Declaration, internationally recognized guidelines, and the privacy of patients was protected by decoding of data according to the privacy regulations of the Orenburg Regional Clinical Oncologic Center (Russia, Orenburg). Written informed consent was obtained from the patient and the protocol was approved by the Institutional Review Board of the Orenburg State Medical University (Russia, Orenburg).

Clinical and pathological data including age, grade, tumour size, lymph node status were retrieved from the routine reports. Information regarding estrogen (ER), progesterone (PR) receptor status and HER-2 status was retrieved from the pathology reports. Tumor size and nodal status were categorized according to the TNM classification of malignant tumors (the 7th edition), and histological grade was evaluated using the Nottingham modification of the Bloom and Richardson histological grading criteria (Robbins et al. 1995). These data are summarized in the Table 1. The average age of the patients was 58.1 ± 10.1 years (from 35 to 75 years, the median—was 57 years). All patients were treated with either modified radical mastectomy (n = 45) or breast-conserving therapy (n = 14), but all with adequate lymph node dissection (at least 10–12 nodes were examined). None of the patients recruited in this study were undergone preoperative chemotherapy or radiotherapy.

**Table 1:** Clinicopathologic characteristics of breast carcinoma cases

Clinicopathologic variables	Number of cases (n)	Percent (%)
Age		
<50	11	18.6
>50	48	81.4
Tumor status (T)		
pT1	21	35.6
pT2	38	64.4
Nodal status (N)		
pN0	30	50.8
pN1	15	25.4
pN2	1	1.7
pN3	13	22.1
Number of lymph nodes		
0	30	50.8
1–3	20	33.9
4–6	6	10.2
>6	3	5.1
Tumour grade		
G1	7	11.9
G2	42	71.2
G3	10	16.9
ER status		
Negative	29	49.1
Positive	30	50.9
PR status		
Negative	38	64.4
Positive	21	35.6
HER2/neu status		
Negative	48	81.4
Positive	11	18.6

### Pathology

The sections (4 mm) were cut from the formalin-fixed paraffin embedded blocks. One section was stained with Mayer’s hematoxylin and eosin (H&E). Histological slides were studied by light microscopy (Optika B-350 microscope, connected to a ScopeTek DCM500 camera, Italy). The number of dilated capillaries (DCs) and CS type-2 were assessed by visual analog way using a 200× magnification (none, single—no more than two in the field of view, and multiple—more than two in the field of view). The presence or absence of local lymphoid infiltrates (LI) and loose fine-fibered connective tissue (LFFCT) in peritumoral stroma was estimated in the same way. All sections were carefully and completely scanned by two of the authors (MS and NP) without knowledge of the clinical and pathological data.

### Immunohistochemistry

Forty five sections were stained with antibody to CD 34. The sections for immunohistochemistry (IGH) were dewaxed and rehydrated by sequential immersion in xylene and graded into ethanol and water. For antigen retrieval the sections were boiling for 10 min in citrate buffer (pH 6) and endogenous peroxidase activity was blocked with 30 mL/L hydrogen peroxide solution. Adjacent slides were incubated at a room temperature with the anti-CD34 (QB–END/10, Novocastra Laboratories Ltd) monoclonal antibodies in diluted at 1:50. The time of antibodies incubation was according to the manufacturer protocol. The visualization system included DAB (UltraVision LP Detection System HRP Polymer and DAB Plus Chromogen) and hematoxylin counterstaining. For negative control sections primary antibody was replaced with phosphate-buffered saline and processed in the same manner.

The slides were briefly scanned at low power, and intra- and peritumoral areas with the highest density of CД34-positive vessels were identified. Intratumoral vessels were defined as those located within the tumor mass. Peritumoral vessels were those located outside of the tumor mass but within 2 mm from the tumor edge. The number of atypical dilated capillaries (ADCs) and CS type-1 were calculated by visual analog way using a 200× magnification (none, single—no more than two in the field of view, and multiple—more than two in the field of view). Microvessels density (MVD) was assessed in accordance with the international consensus on the methodology and criteria for quantitative evaluation of angiogenesis in human solid tumors (Vermeulen et al. 2002). MVD was determined by counting the number of CД34-positive normal microvessels (MVs) in five high-power (200×) fields in the selected ‘hot-spot’ areas, and the mean values of vessel counts were obtained. A single, countable microvessel was defined as any brown-stained endothelial cell (or cluster) clearly separated from the adjacent microvessels.

### Statistics

Statistical analysis was performed using the Statistica 6.0 software. The MVD were expressed as mean ± SD. Kruskal–Wallis and Mann–Whitney U nonparametric tests were used to compare the value of MVD. The correlations between different data were evaluated using nonparametric Spearman’s rank correlation or gamma correlation. Chi square tests were carried out to analyze the difference of distribution among the categorized data. A value of P < 0.05 was considered statistically significant.

## Results

The study showed that in BC the tumor vessels are heterogeneous in morphology and clinical significance. We identified the following types of tumor vessels:

### The normal microvessels (MVs) of peritumoral and intratumoral stroma

**Fig. 1:**
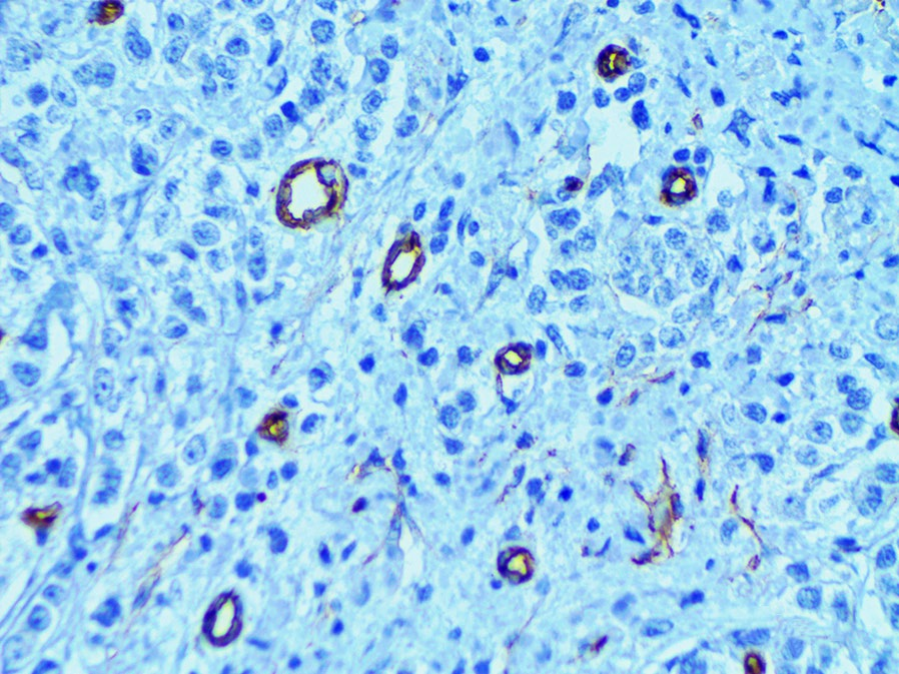
The normal microvessels of intratumoral stroma, immunoperoxidase staining with the anti-CD34 monoclonal antibody, ×400

These vessels were presented by the capillaries with diameter of 5–40 μm. The endothelium lining of such vessels had a flat hyperchromic nucleus. Cytoplasm of endothelial cells uniformly and intensively was being colored by CD34 marker and had clear contours (Fig. 1).

### The dilated capillaries (DCs) of peritumoral stroma

**Fig. 2:**
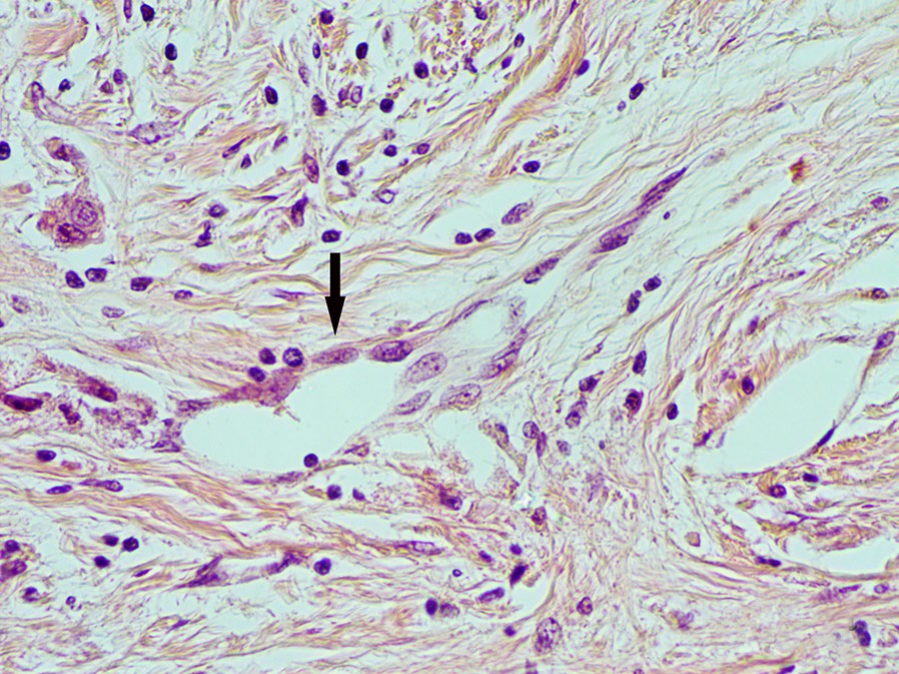
The dilated capillary in peritumoral stroma (*arrow*), H&E stain, ×400

**Fig. 3:**
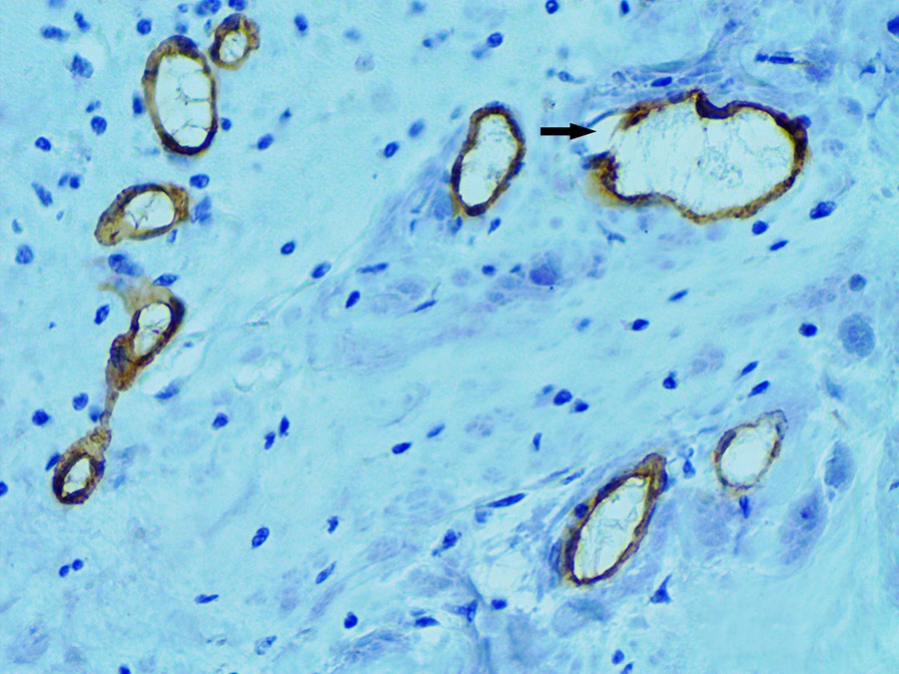
The dilated capillary in peritumoral stroma (*arrow*), immunoperoxidase staining with the anti-CD34 monoclonal antibody, ×400

**Fig. 4:**
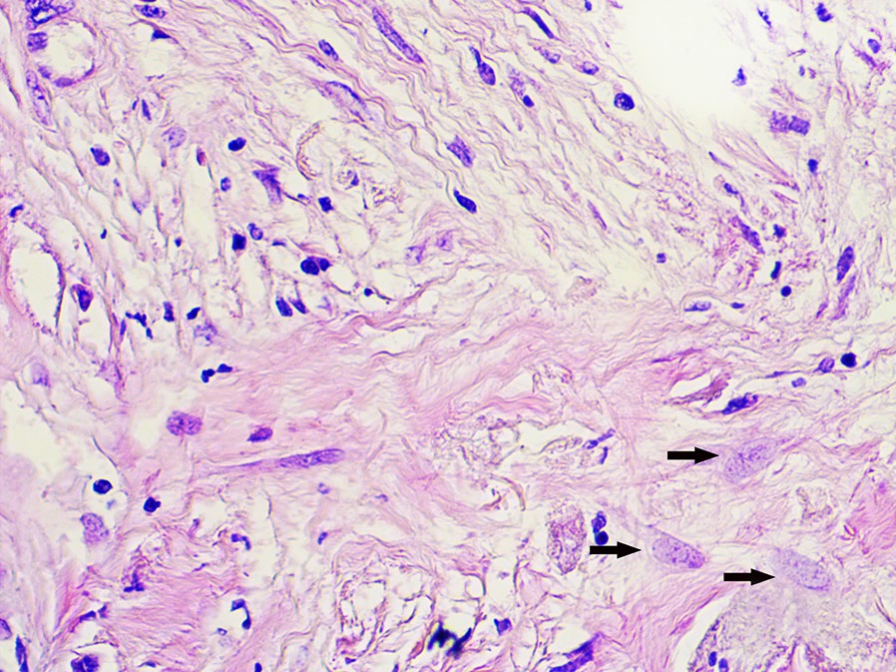
A loose fine-fibered connective tissue with fibroblasts (*arrows*) in peritumoral stroma, H&E stain, ×400

The large vessels were often of rounded or oval shape with a diameter of more than 40 microns (average diameter 87.8 ± 64.4 microns). A distinguishing feature of such vessels was that the cells with large, pale nuclei with fine-netted chromatin structure took part in their formation (Fig. 2). The cytoplasm of the lining cells was being evenly stained by CD34 and had the clear contours (Fig. 3). The presence of the described vessels was often associated with a characteristic structure of peritumoral stroma presented by a LFFCT being rich by fibroblasts (Fig. 4).

### The atypical dilated capillaries (ADCs)

**Fig. 5:**
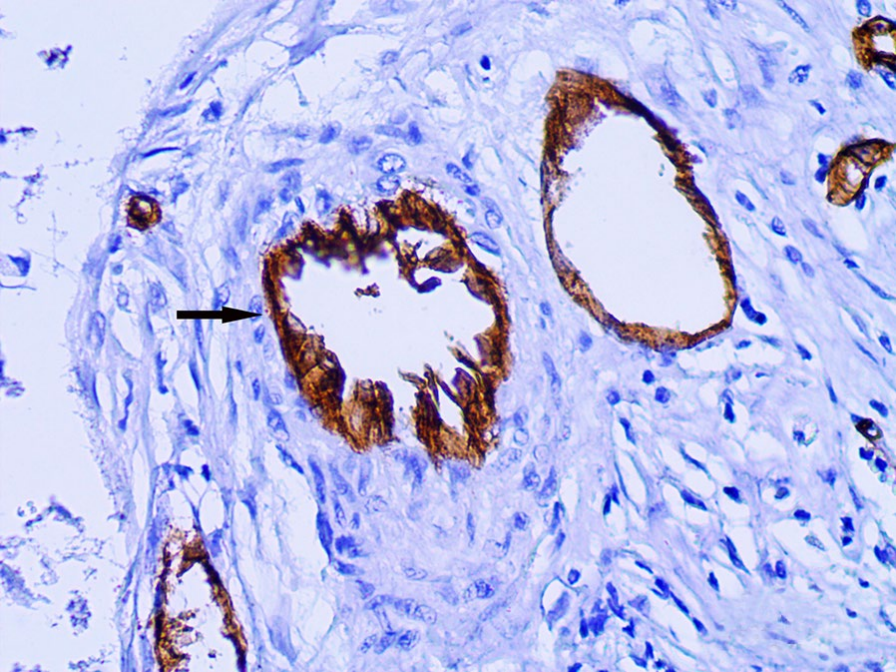
The atypical dilated capillary  (*arrow*) with chaotic arrangement of the endothelial cells.  Their cytoplasm has an uneven surface with a number of protuberances, immunoperoxidase staining with the anti-CD34 monoclonal antibody, ×400

**Fig. 6:**
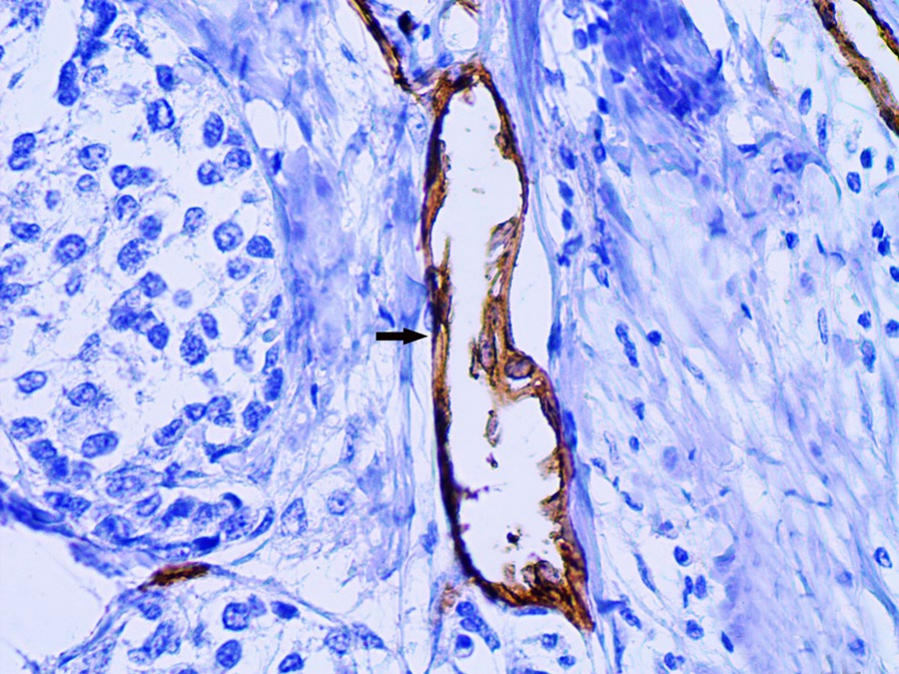
The atypical dilated capillary (*arrow*). The vessel has irregular shape and chaotic arrangement of the endothelial cells, immunoperoxidase staining with the anti-CD34 monoclonal antibody, ×400

**Fig. 7:**
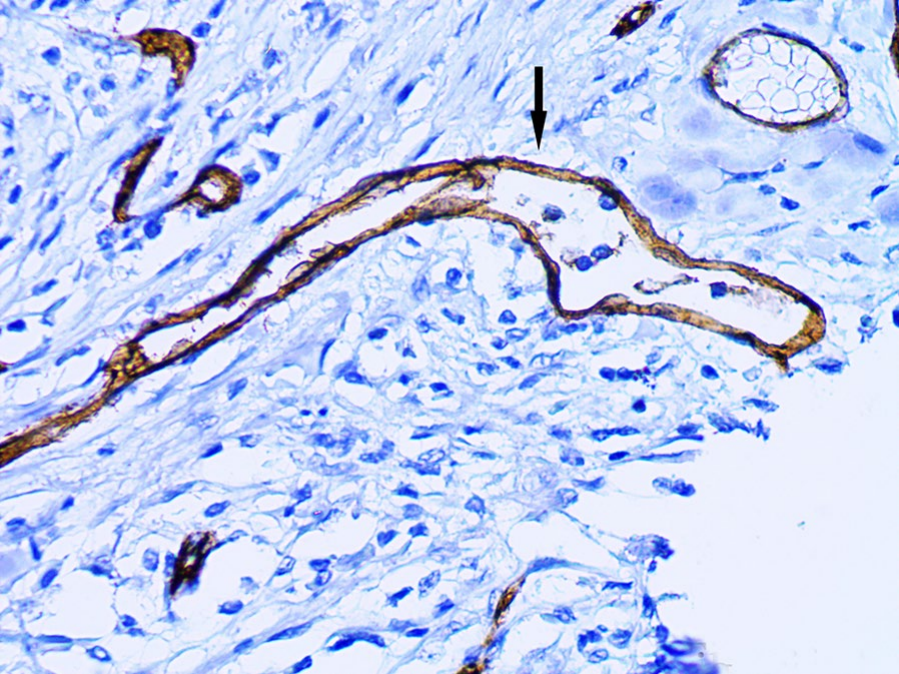
The atypical dilated capillary (*arrow*) with tumor emboli in the  lumen, immunoperoxidase staining with the anti-CD34 monoclonal antibody, ×400

**Fig. 8:**
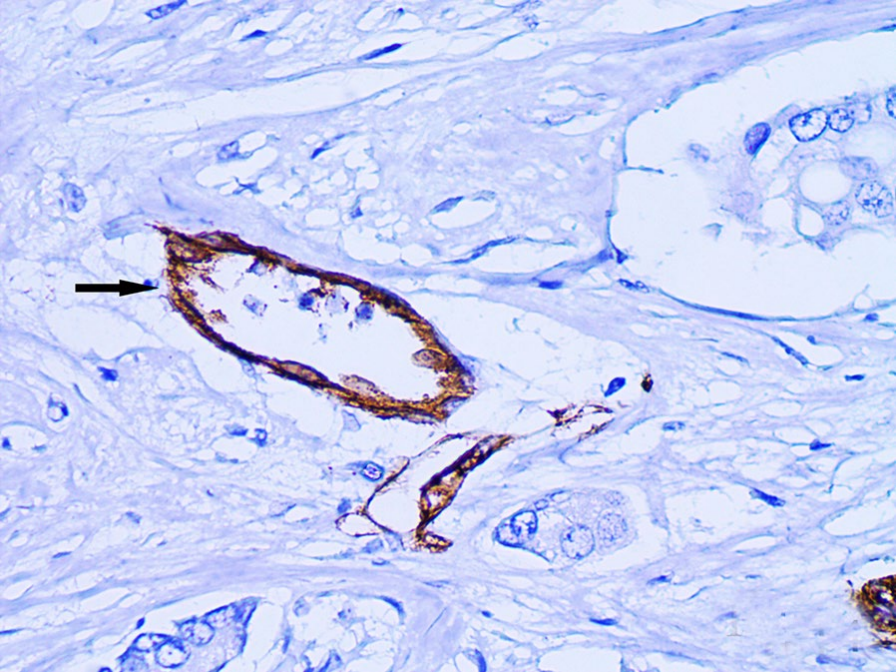
The atypical dilated capillary (*arrow*) located in the intratumoral stroma. Several tumor cells are observed in the lumen of vessel, immunoperoxidase staining with the anti-CD34 monoclonal antibody, ×400

The ADCs were presented the vessels of irregular shape with a diameter of 40 microns or more and with the marked atypia of the lining endothelial cells. These vessels were observed mainly in the intratumoral stroma. A characteristic feature of the described vessels was a chaotic arrangement of the endothelial cells. The cytoplasm of the lining cells was unevenly stained by CD34 and had an uneven surface with a number of protuberances, so that the contours of the vessels seemed to be indistinct (Figs. 5, 6). In the lumen of such vessels the tumor emboli were often being defined (Figs. 7, 8).

### The “cavitary” structures type-1 (CS type-1)

**Fig. 9:**
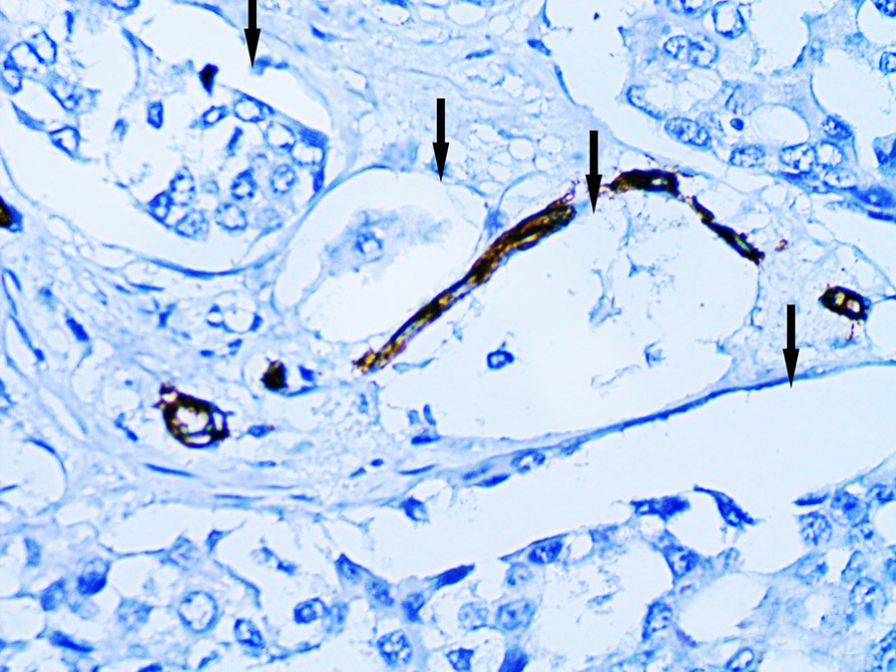
The “cavitary” structures type-1 (*arrows*) without endothelial lining, immunoperoxidase staining with the anti-CD34 monoclonal antibody, ×400

**Fig. 10:**
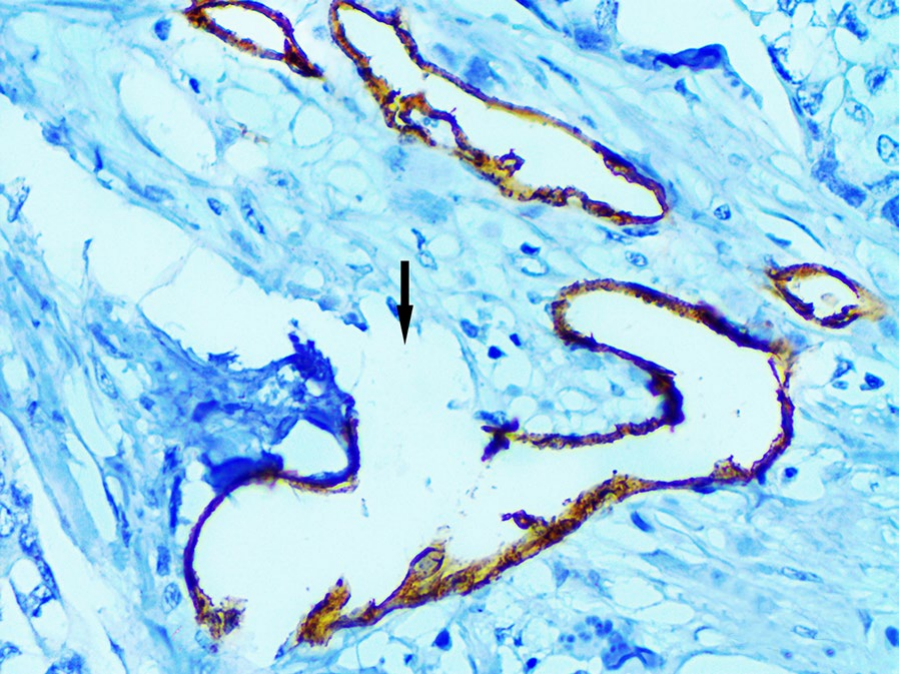
The “cavitary” structure type-1 (*arrow*) with partial endothelial lining, immunoperoxidase staining with the anti-CD34 monoclonal antibody, ×400

**Fig. 11:**
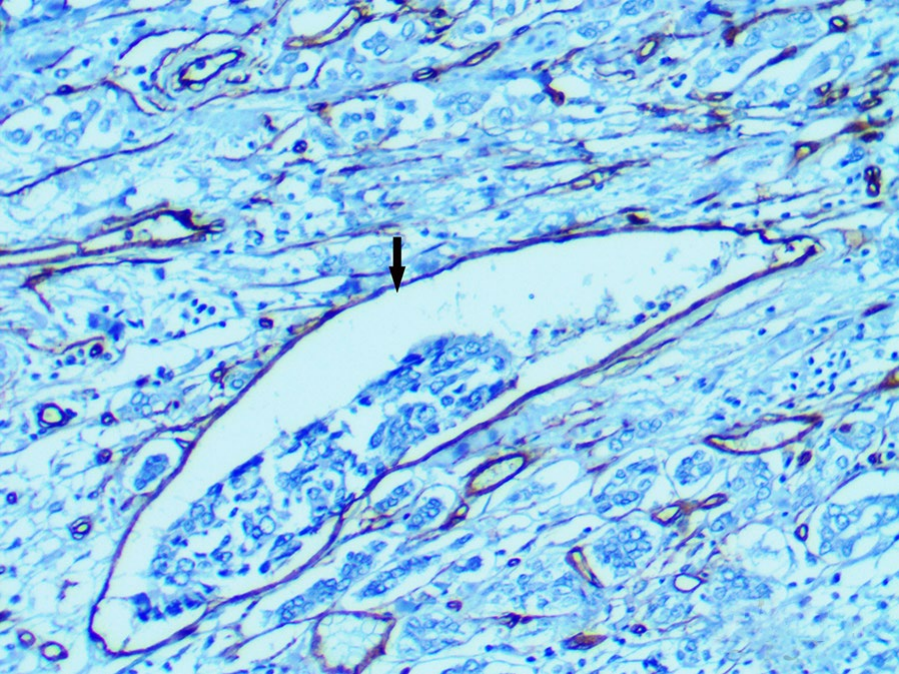
The “cavitary” structure type-1 (*arrow*) with full endothelial lining, immunoperoxidase staining with the anti-CD34 monoclonal antibody, ×400

**Fig. 12:**
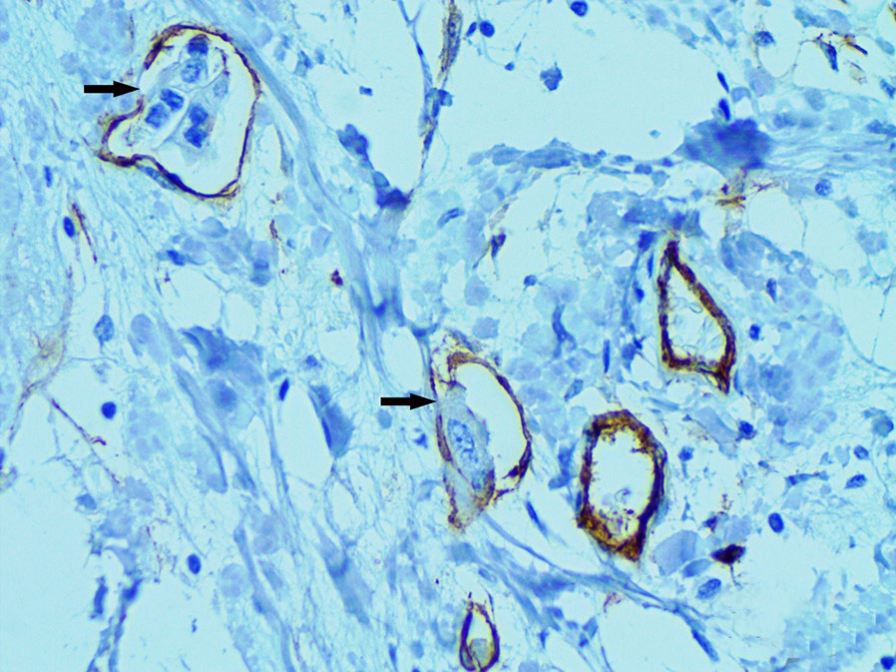
Several “cavitary” structures type-1 (*arrows*) with full endothelial lining, immunoperoxidase staining with the anti-CD34 monoclonal antibody, ×400

As it had been previously described, the CS with partial endothelial lining were the main feature of the “cavitary” angiogenesis type-1 (Senchukova et al. 2015). In BC the CS without endothelial lining (Fig. 9), the CS with partial endothelial lining (Fig. 10) and the CS with full endothelial lining (Figs. 11, 12) were observed in intratumoral and more rarely in peritumoral stroma. As in ADC the cytoplasm of the endothelial cells in CS type-1 with partial endothelial lining was also unevenly stained by CD34 and had an uneven surface with a number of protuberances.

### The “cavitary” structures type-2 (CS type-2)

**Fig. 13:**
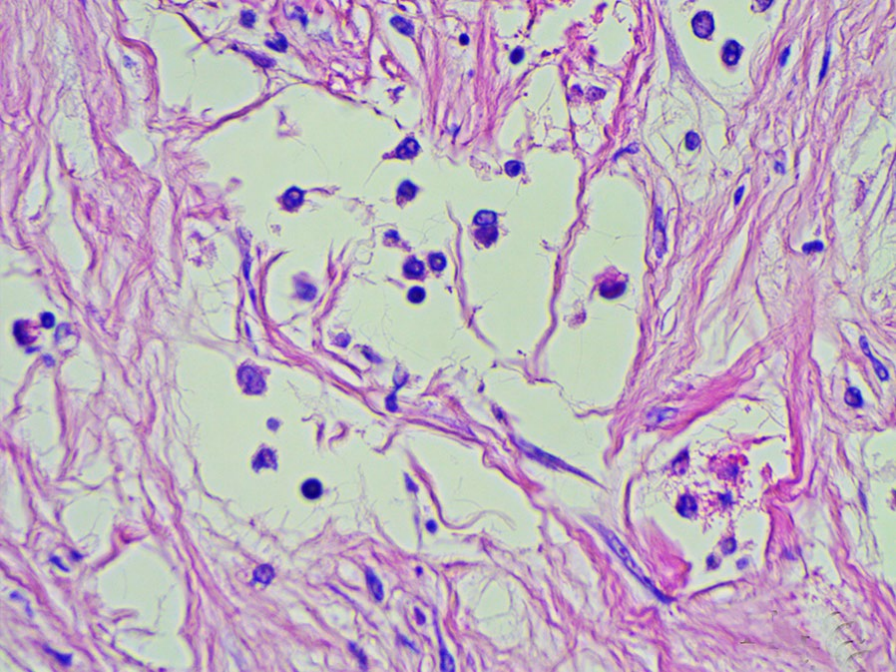
A characteristic cellular structure of loose fine-fibered connective tissue in peritumoral stroma, H&E stain, ×400

**Fig. 14:**
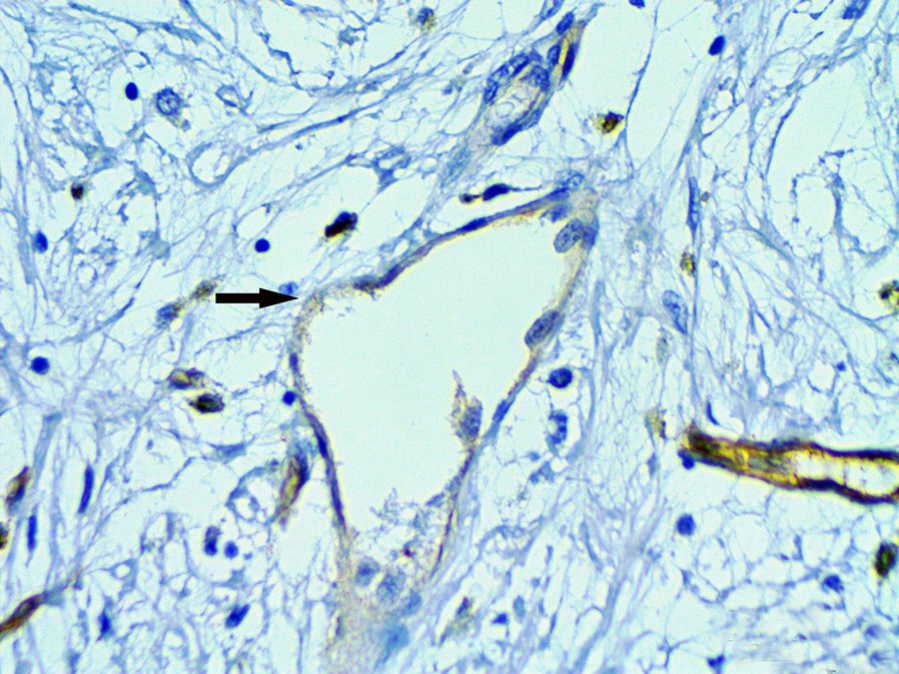
The “cavitary” structure type-2 with endothelial lining having a very weak expression of CD34 (6b), immunoperoxidase staining with the anti-CD34 monoclonal antibody, ×400

We observed CS type-2 only in the above-described LFFCT of peritumoral stroma. In these cases the LFFCT had a distinctive cellular structure (Fig. 13). The CS type-2 had a correct form, often oval. Some CS type-2 had the endothelial lining the cells of which were very poorly expressed CD34 (Fig. 14).

### The association of different types of tumor vessels with the clinical and morphological characteristics of breast cancer

#### The normal MVs of peritumoral and intratumoral stroma

MVD in peritumoral and intratumoral stroma was 9.8 ± 3.9 and 9.8 ± 3.2 on area unit, respectively. The Spearman rank correlation analysis (ρ) showed that the MVD in peritumoral stroma correlated with the presence of lymph node metastases (σ = −0.310, t = −2.14, p = 0.04). The MVD in peritumoral stroma was higher in the absence of metastasis than in their presence (9.34 ± 3.00 and 10.22 ± 4.84, p = 0.04, respectively, Mann–Whitney U Test). The correlations of MVD in intratumoral stroma with clinical characteristics of BC were not identified.

#### The DCs of peritumoral stroma

The single DCs were detected in 26 patients (44.1 %), the multiple - in 26 patients (44.1 %), the DCs were absent in 7 (11.8 %). The gamma correlation coefficient test (gamma) showed that the number of DCs correlated with the presence of LI (γ = 0.489, Z = 3.66, p = 0.0002) and LFFCT (γ = 0.563, Z = 3.54, p = 0.0004) in the peritumoral stroma, the presence of CS type-1 with partial endothelial lining (γ = 0.574, Z = 2.99, p = 0.003), the presence of CS type-2 (γ = 0.522, Z = 3.14, p = 0.002), the presence of ADCs (γ = 0.533, Z = 3.35, p = 0.0008) and with the presence of tumor emboli in vessels (γ = 0.520, Z = 2.83, p = 0.005). It was not associated with clinical characteristics of BC.

The multiple DCs were more often observed in the presence of LI (in 29.1 and 69.6 % cases in the presence and absence of LI, respectively, χ^2^ = 10.22, p = 0.04) and LFFCT (in 53.8 and 25 % cases in the presence and absence of LFFCT, respectively, χ^2^ = 7.14, p = 0.03, respectively) in peritumoral stroma.

In the presence of multiple DCs, the CS type-1 with partial endothelial lining, CS type-2, ADCs and tumor emboli in vessels were more often revealed (Table 2).

**Table 2:** The presence of atypical dilated capillaries, CS type-1 with partial endothelial lining, CS type-2 and tumor emboli in vessels depending on the number of dilated capillaries in peritumoral stroma

	The number of dilated capillaries						p value
	No		Single		Multiple		
	n	%	n	%	n	%	
Atypical dilated capillaries							
No	2	40.0	7	33.3	1	5.3	0.13
Single	2	40.0	8	38.1	7	36.8	
Multiple	1	20.0	6	28.6	11	57.9	
CS type-1							
Absent	2	40.0	9	42.9	2	10.5	0.07
Present	3	60.0	12	57.1	17	89.5	
CS type-2							
Absent	6	85.7	20	66.9	11	42.3	0.02
Present	1	14.3	6	23.1	15	57.7	
Tumor emboli							
Absent	3	60.0	10	47.6	4	21.0	0.12
Present	2	40.0	11	52.4	15	78.9	

### The ADCs

The presence of ADCs was associated with the grade of BC (γ = 0.525, Z = 2.40, p = 0.003), estrogen (ER) and progesterone (PR) receptor status (γ = −0.325, Z = −2.01, p = 0.045), the presence of CS type-1 with partial endothelial lining (γ = 0.753, Z = 5.27, p < 0.00001), the presence of CS type-2 (γ = 0.717, Z = 4.13, p = 0.00004) and with the presence of tumor emboli in vessels (γ = 0.647, Z = 3.81, p = 0.0002).

The multiple ADCs were significantly more often in grade G2–G2 (in 0, 43.8 and 50 % cases in G1, G2 and G3, respectively, χ^2^ = 11.28, p = 0.03), in negative RE status (in 60.0 and 15.0 % cases, in negative and positive RE status, respectively, χ^2^ = 9.69, p = 0.008), in negative RP status (in 47.1 % и 18.2 % cases, in negative and positive RP status, respectively, χ^2^ = 2.98, p = 0.22).

In the presence of multiple ADCs the CS type-1 with partial endothelial lining, CS type-2 and tumor emboli in vessels were revealed significantly more often (Table 3).

**Table 3:** The presence of CS type-1 with partial endothelial lining, CS type-2 and tumor emboli in vessels depending on the number of atypical dilated capillaries

	The number of atypical dilated capillaries						p value
	No		Single		Multiple		
	n	%	n	%	n	%	
CS type-1							
No	7	70.0	4	23.5	2	11.1	0.0004
Single	3	30.0	10	58.8	5	27.8	
Multiple	0	0	3	17.7	11	61.1	
CS type-2							
No	3	30.0	8	47.1	5	27.8	0.14
Single	5	50.0	5	29.4	3	16.7	
Multiple	2	20.0	4	23.5	10	55.6	
Tumor emboli							
Absent	7	70.0	7	41.2	3	16.7	0.02
Present	3	30.0	10	58.8	15	83.3	

### The “cavitary” structure type-1 (CS type-1)

The number of CS type-1 with partial endothelial lining also correlated with ER (γ = −0.622, Z = −3.08, p = 0.002) and PR status (γ = −0.645, Z = −3.12, p = 0.002) and with a presence of tumor emboli in vessels (γ = 0.515, Z = 2.92, p = 0.004).

The CS type-1 were more often observed in negative RE status (in 84.0 % and 55.0 % cases in negative and positive RE status, respectively, χ^2^ = 4.55, p = 0.03) and in negative RP status (in 79.4 and 45.5 % cases, in negative and positive RP status, respectively, χ^2^ = 4.66, p = 0.03).

In the cases of multiple CS type-1 the tumor emboli in vessels were more often revealed (in 46.2, 55.6 and 85.7 % cases, in the absence of CS type-1, single and multiple ones, respectively, χ^2^ = 5.05, p = 0.08).

### The “cavitary” structures type-2 (CS type-2)

The presence of CS type-2 were associated with the presence of LFFCT in the peritumoral stroma (γ = 0.983, Z = 11.19, p < 0.00001), with the positive Her2/new status (γ = 0.680, Z = 3.66, p = 0.0002) and with the presence of tumor emboli in vessels (γ = 0.441, Z = 2.48, p = 0.01).

The CS type-2 were more often observed in the presence of LFFCT in peritumoral stroma (in 97.4 and 0 % cases in the presence and absence of LFFCT, respectively, χ^2^ = 54.75, p < 0.00001) and in positive Her2/new status (in 100.0 and 56.3 % cases in the positive and negative Her2/new status, respectively, χ^2^ = 9.69, p = 0.008). In the presence of CS type-2 the tumor emboli in vessels were more often revealed (in 43.7, 69.2 and 75.0 % cases in the absence of CS type-2, single and multiple ones, respectively, χ^2^ = 3.71, p = 0.16).

## Discussion

A large amount of studies testified that the vessels in the tumor are heterogeneous and differ in origin, morphology, clinical significance and sensitivity to anti-angiogenic therapy (Baluk et al. 2005; Luukkaa et al. 2007; Mucci et al. 2009; Taverna 2009; Birau et al. 2012; Fukumura et al. 2010; Nagy and Dvorak 2012; Mikalsen et al. 2013). In this study we decided to investigate the morphological features of tumor vessels in BC. The obtained data allowed us to identify the several types of tumor vessels differing both in morphology and clinical relevance: the normal MVs, DCs of peritumoral stroma, ADCs and the CS type-1 and type-2 relating to “cavitary” type of angiogenesis (Senchukova and Kiselevsky 2014).

The normal MVs were presented the capillaries of 5–40 microns in diameter. Their endothelial lining was intensively and uniformly being stained by CD34. MVD was absolutely identical in peri- and intratumoral stroma. However, MVD in peritumoral stroma was slightly higher in the absence of metastases in lymph nodes than in the presence of them (p = 0.04), that was not entirely clear. We did not identify the relation of MVD with other clinical characteristics of BC. These results contradict the data of other authors that found an association of MVD with clinical characteristics and prognosis of BC (Li et al. 2006; Popiela et al. 2008; Fernández-Guinea et al. 2013; Li et al. 2013). We believe that the reason for these contradictions are due to the fact that in contrast to the above-mentioned authors in calculating of MVD we took into account only the normal microvessels, while the prognosis was associated with other types of tumor vessels.

The DCs were revealed in the peritumoral stroma in 88.2 % of cases. A distinguishing feature of such vessels was that the cells with large, pale nuclei with fine-netted chromatin structure took part in their formation. The cytoplasm of lining cells was uniformly being stained by marker and had clear contours. The localization of vessels in peritumoral stroma and the lack of red blood cells in their lumen testified in favor of the fact that these vessels were lymphatic ones. But for a more precise determination of their origin the additional research with using the special markers such as D2-40 is needed.

The presence of these vessels was not associated with clinical characteristics of BC but their number was positively correlated with the number of ADCs, CS type-1 and CS type-2 as well as with the presence of LFFCT and LI in the peritumoral stroma. We believe that this fact is the indicative of the existence of common mechanisms in the formation of described vessels. And these mechanisms have its origin in the close relationship of the processes of stroma formation, angiogenesis, lymphangiogenesis and inflammation (Ebelt et al. 2013; Costa et al. 2014; Sharon et al. 2015). Furthermore, the features of the described vessels allow us to suggest that angioblasts should participate in their formation. Their involvement in tumor angiogenesis has been described by many researchers (Sussman et al. 2003; Richter-Ehrenstein et al. 2007; Naik et al. 2008).

It should be noted that one of the most important prognostic criteria in BC comparable to the status of the lymph nodes is lymphovascular invasion (LVI). LVI is the presence of tumor cells in blood and/or lymphatic vessels. A large number of studies testifies that this indicator is associated with the size of the tumor with the presence of lymph node metastases and prognosis of BC (Kato et al. 2003; Lee et al. 2006; Arnaout-Alkarain et al. 2007; Gudlaugsson et al. 2011; Rakha et al. 2012; Mohammed et al. 2013). At the St. Gallen meeting in 2005, LVI was recognised as a prognostic factor for node-negative patients (Goldhirsch et al. 2005). However, we emphasize that LVI is not an independent prognostic factor of BC. LVI is also associated with other prognostic factors including tumor size, grade and loco-regional lymph node involvement, expression of VEGF-C (Lee et al. 2006; Li et al. 2006; Freedman et al. 2012). In some studies the relationship of LVI with the RE status was noted, however, the connection of LVI with the HER2 status was not marked (Marinho et al. 2008; Ragage et al. 2010; Song et al. 2011; Lee et al. 2011; Mohammed et al. 2013).

Despite the fact that the LVI has long been used as an important prognostic factor, the mechanisms of the tumor emboli formation in the vessels are still unclear. Using the notion of “metastatic cascade” (Engers and Gabbert 2000; Weigelt et al. 2005) it is extremely difficult to explain how the large portions of the tumor tissue are placed into the lumen of vessels. However, this phenomenon is well explained by the hypothesis of “cavitary” type of angiogenesis, proposed by us earlier (Senchukova and Kiselevsky 2014; Senchukova et al. 2015). According to this hypothesis, the formation of blood vessels can occur due to the formation of the CS in tumor stroma, being then lined by the endothelium and merged into the blood vessels of the organ. We have noted two main types of the CS formation: formation of CS at the expense of abruption of layers of epithelial cells from their underlying foundation (CS type-1) and the formation of CS directly in the tumors stroma at the expense of active processes of formation and lysis of its elements (CS type-2). We believed that the formation of the CS type-1 was directly associated with the phenomenon of stroma retraction that had been described previously (Acs et al. 2007, 2012).

The analysis of our data showed that in BC the presence of LVI was closely associated with the CS type-1 with the partial endothelial lining (p = 0.004) and with the ADCs (p = 0.0002). We believe that most probably the ADCs have direct relation to “cavitary” angiogenesis type-1 as these two factors were closely correlated with each other (p < 0.00001). The ADCs and CS type-1 was most often observed in the ER and PR negative tumors. At the same time, their presence was not associated with Her 2 new status. It was difficult to judge the origin of ADCs because in our study we used the marker of CD34 staining both the lymph and blood vessels. Considering that the erythrocytes were often defined in the lumen of ADCs and taking into account the data of different authors showing that the intratumoral lymphatic vessels were extremely rare (Schoppmann et al. 2004; Kato et al. 2005; Gudlaugsson et al. 2011), we think that ADCs is likely to have a bearing on the blood vessels. More precisely the affiliations of these vessels may be determined using the specific methods of lymphatic vessels staining, e.g. CD2-40. The research of this kind seems to be important since the clinical evidence testifies that the hematogenous and lymphogenous path of metastasis are independent factors associated with the progression of BC.

As for the CS type-2, of particular interest is the relationship of these vessels with the LFFCT (p < 0.00001) and Her2/new status (p = 0.0002). These data can have both the theoretical and prognostic value since they show that the Her2/new status is closely linked with the stroma formation and with and angiogenesis as well. Also it was noted a close link of Her2/new status with the presence of tumor emboli in the vessels (P = 0.004) but its connection with CS type-1 was not observed.

Thus, these data indicate that in BC the tumor vessels are heterogeneous in morphology and clinical significance. The most significant structures in terms of prognosis are CS type-1 and type-2 and ADCs. The CS type-1 and ADCs are associated with the presence of tumor emboli in the vessels (LVI) and with the negative ER and PR status, and the CS type-2—with positive Her2/new status and with the presence of LFFCT. The obtained data may be of theoretical and practical interest. We believe that further studies are required for the understanding of angiogenesis mechanisms in breast cancer.

## Abbreviations

BC: breast cancer
MVs: microvessels
DCs: dilated capillaries
ADCs: atypical dilated capillaries
CS: “cavitary” structures
MVD: microvessel density
ER: estrogen receptor
PR: progesterone receptor
HER-2: human epidermal growth factor receptor 2
GC: gastric cancer
LFFCT: loose fine-fibered connective tissue
